# Cancer-Induced Metabolic Rewiring of Tumor Endothelial Cells

**DOI:** 10.3390/cancers14112735

**Published:** 2022-05-31

**Authors:** Jacopo Lidonnici, Massimo M. Santoro, Roxana E. Oberkersch

**Affiliations:** Laboratory of Angiogenesis and Cancer Metabolism, Department of Biology, University of Padova, 35122 Padova, Italy; jacopolidonnici@gmail.com (J.L.); massimo.santoro@unipd.it (M.M.S.)

**Keywords:** tumor vasculature, tumor endothelial cells, endothelial metabolism in cancer

## Abstract

**Simple Summary:**

Angiogenesis, the formation of new blood vessels from preexisting ones, is a complex and demanding biological process that plays an important role in physiological, as well as pathological conditions, including cancer. During tumor growth, the induction of angiogenesis allows tumor cells to grow, invade, and metastasize. Recent evidence supports endothelial cell metabolism as a critical regulator of angiogenesis. However, whether and how tumor endothelial cells rewire their metabolism in cancer remains elusive. In this review, we discussed the metabolic signatures of tumor endothelial cells and their symbiotic, competitive, and mechanical metabolic interactions with tumor cells. We also discussed the recent works that may provide a rationale for attractive metabolic targets and strategies for developing specific therapies against tumor angiogenesis.

**Abstract:**

Cancer is a leading cause of death worldwide. If left untreated, tumors tend to grow and spread uncontrolled until the patient dies. To support this growth, cancer cells need large amounts of nutrients and growth factors that are supplied and distributed to the tumor tissue by the vascular system. The aberrant tumor vasculature shows deep morphological, molecular, and metabolic differences compared to the blood vessels belonging to the non-malignant tissues (also referred as normal). A better understanding of the metabolic mechanisms driving the differences between normal and tumor vasculature will allow the designing of new drugs with a higher specificity of action and fewer side effects to target tumors and improve a patient’s life expectancy. In this review, we aim to summarize the main features of tumor endothelial cells (TECs) and shed light on the critical metabolic pathways that characterize these cells. A better understanding of such mechanisms will help to design innovative therapeutic strategies in healthy and diseased angiogenesis.

## 1. Introduction

### 1.1. Blood Vessel Formation

The vascular tree is a dense network of closed blood vessels that branch out along the entire body. This vascular network carries oxygen and nutrients to the tissues and drains waste products [1]. Although blood vessels in our body are very different in shape and size, all share the same structure, such as a monolayer of endothelial cells (ECs) that delimits their walls. Under physiological conditions, an adult vasculature is composed of quiescent ECs that, under specific circumstances, can reactivate cell proliferation and migration, becoming activated proliferating ECs. This reactivation and the formation of new blood vessels from pre-existing vessels, is a process known as angiogenesis [2]. This process occurs in physiological as well as pathological conditions [3]. In addition to embryonic development, angiogenesis takes place in adult tissues such as during wound repair and the female reproductive cycle. Angiogenesis can also occur during tumor growth, and the induction of angiogenesis within solid tumors is a cancer hallmark [4]. The discovery of the vascular endothelial growth factors (VEGFs) [5], and therefore the existence of surface receptors capable of binding such ligands, which, due to their surface localization, are easily druggable, has led to the development of biological anti-angiogenic drugs capable of targeting these receptors [6]. Nevertheless, the addition of these drugs to chemotherapy has shown modest effects on patient survival: tumors often escape treatments and regain angiogenic capacity or gain access to the bloodstream by co-opting non-malignant vessels within themselves [7], resulting in a worse prognosis. This led to a paradigm shift in anti-angiogenic therapeutic strategies, from starving the tumor by destroying tumor blood vessels to “normalize” the tumor vasculature in order to better deliver anti-tumor drugs [8]. Another piece of the puzzle came from the discovery that tumor ECs are highly dependent on metabolic processes, such as glycolysis [9]. This observation opened the innovative hypothesis of being able to target the ECs’ metabolism to normalize the vasculature of tumors and thus prevent their growth [10,11]. Understanding tumor EC metabolism is mandatory to better design therapeutic strategies.

### 1.2. Tumor Vasculature

Tumor cells shape the tumor microenvironment (TME) [12]. Rapidly growing solid tumors (e.g., carcinomas) incorporate various cell types such as fibroblasts and immune cells into their parenchyma to secrete factors that confer survival benefits [13]. As the volume of the tumor grows, the diffusion of oxygen (O_2_) decreases making the center of mass hypoxic and necrotic. Low oxygen levels activate the hypoxia inducible factor (HIF), which in turn leads to the expression of growth factors such as VEGF that trigger angiogenesis [14]. This implicates that tumor mass volume is critical for the angiogenic switch [15,16]. The uncontrolled hyper-release of pro-angiogenic factors causes abnormal vessel growth. Unlike the normal vasculature, which has a high hierarchical organization in vascular beds (veins, arteries, and capillaries), the tumor vasculature appears as a disorganized tangle of vessels [17] often with a blind end. Tumor endothelial cells (TECs), unlike normal endothelial cells (NECs), can grow in multilayers along the vessel wall [18]. Moreover, the reduction of cellular junctions [19], the decrease in mural cells (pericytes and smooth muscle cells) [20], and a strong compromise of the basement membrane determine vessel leakage. This generates an increase in the interstitial fluids pressure that in turn causes the collapse of the less resistant vessels [21], while the blood flow within the tumor vessels becomes chaotic [22]. The microhemorrhages of these vessels allow the deposition of fibrin, which acts as an anchor for the tumor cells [23]. In addition, the increase in stiffness of the parenchyma, in part, due to vessel leakage, makes the tumor poorly perfused and resistant to chemotherapy treatments.

The vascular supply required by cancer cells to support their growth can be provided by the hijacking of normal vessels from surrounding tissues, a process known as co-option [7], or by the formation of new vessels within the tumor. The classic sprouting angiogenesis model foresees that in response to pro-angiogenic cues, such as VEGF, FGF, ANG-2, and several other chemokines, the basement membrane is locally degraded by metalloproteases (MMPs) and the surrounding pericytes detach from the vessel wall. This allows the ECs to resume proliferative activity. An EC engages an invasive phenotype, extending filopodia and lamellipodia towards the chemokine gradient, and driving the sprouting of the extending blood vessel [24].

### 1.3. Molecular Aspects of Tumor Endothelial Cells (TECs)

**Figure 1:**
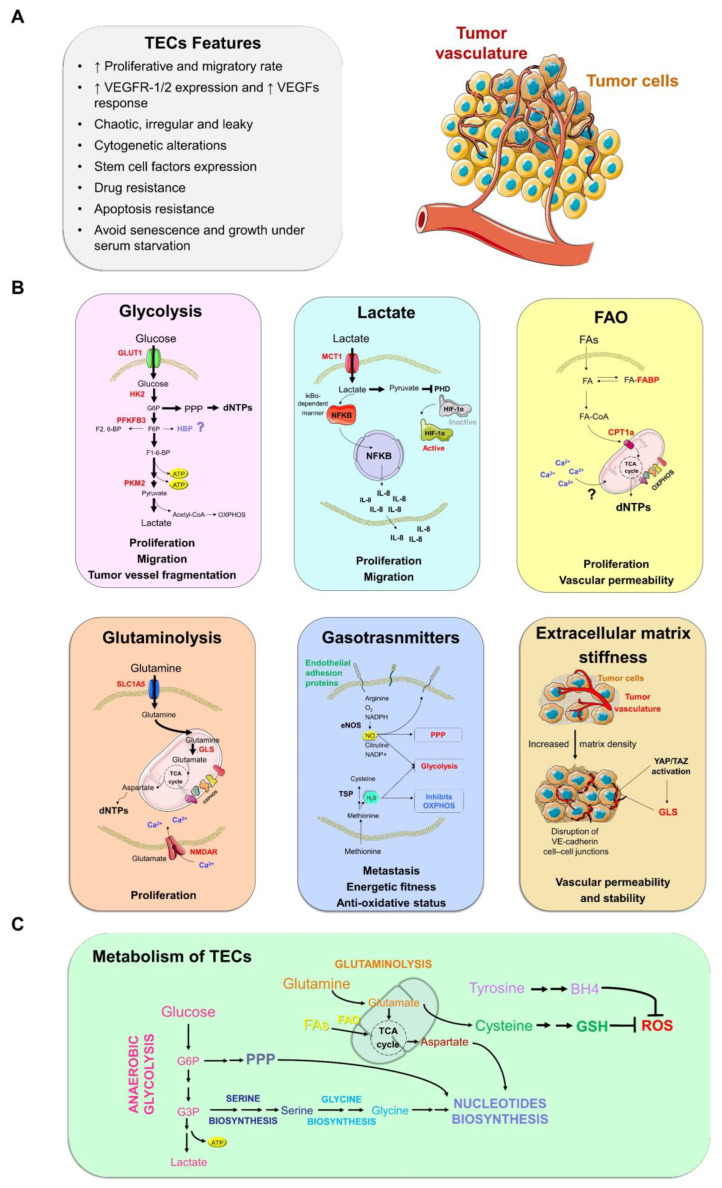
Overview of tumor endothelial cell (TEC) metabolism. (**A**) Phenotypically, TECs show a high replication rate coupled to invasiveness, over-expression of receptors, and pro-angiogenic growth factors such as the vascular endothelial growth factor/vascular endothelial growth factor receptor 2 (VEGF/VEGFR2) axis, cytogenetic alterations, stemness and chemotherapy resistance, the ability to grow in serum-free media, and reduced senescence and apoptosis. (**B**) The main metabolic pathways reported so far in the remodeling of endothelial cell metabolism in cancer context. Proliferating TECs strongly depend on glycolysis and glucose uptake to produce adenosine triphosphate (ATP). Glycolysis provides the energy necessary to guide TEC proliferation and migration; lactate present in tumor microenvironment is imported into TECs through monocarboxylate transporter 1 (MCT-1) and can fuel vessel sprouting by two ways. By activating the nuclear factor kappa-light-chain-enhancer of activated B cells (NF-kB) pathway that up-regulates interleukin 8 (IL-8) and leads to the IL-8 self-stimulation, or by converting into pyruvate that inhibits prolyl hydroxylase (PHD2) activating hypoxia-inducible factor 1 alpha (HIF1α); Fatty acids oxidation (FAO) provides intermediate metabolites and biomass to support sprouting vessels. The EC-specific deletion of both carnitine palmitoyltransferase 1a and 2 (CPT1a and CPT2, respectively), genes that regulate the carnitine shuttle, cause vessel leakage; gasotransmitters such as hydrogen sulfide (H_2_S) and nitric oxide (NO) play a pleiotropic role by altering the proteome of cells and their pro-angiogenic action affects metabolism; extracellular matrix stiffness promotes tumor vessels formation by yes-associated protein (YAP)/transcriptional coactivator with PDZ-binding motif (TAZ) activation and leads to angiogenesis. The enzymes/transporters directly involved in tumor angiogenesis are highlighted in red. (**C**) Metabolic interaction within individual pathways in TECs. The Figure was partly generated using Servier Medical Art.Servier Medical Art by Servier is licensed under a Creative Commons Attribution 3.0 Unported License (https://creativecommons.org/licenses/by/3.0/, accessed on 30 March 2022). ↑: High. GLUT1: glucose transporter 1; HK2: hexokinase 2; PPP: pentose phosphate pathway; HBP: hexosamine biosynthetic pathway; G6P: glucose-6-phosphate; PFKFB3: 6-phosphofructo-2-kinase/fructose-2,6-biphosphatase 3; F2,6-BP: Fructose 2,6-bisphosphate; F6P: fructose 6-phosphate; F1,6-BP: fructose 1,6-bisphosphate; OXPHOS: oxidative phosphorylation; FAs: fatty acids; FABP: fatty acid binding protein; TCA cycle: tricaboxylic cycle; dNTPs: deoxyribonucleoside triphosphates; SLC1A5: solute carrier family 1 member 5; GLS: glutaminase; NMDAR: N-methyl-D-aspartate receptor; eNOS: endothelial nitric oxide synthase; ROS: reactive oxygen species; BH4: tetrahydrobiopterin; GSH: glutathione.

The structural differences of the tumor vessels are reflected both at the cellular and molecular level on the morphological and physiological alterations of the ECs (Figure 1A).

Murine TECs isolated from different human tumor xenografts showed a high proliferative and migratory rate, a greater response to VEGFs, and a higher expression of VEGF receptors-1/2 compared to NECs [25]. Furthermore, these cells show cytogenetic alterations [26] and overexpress stem cell factors such as Sca-1 (stem cell antigen-1) [25] and MDR1 (multidrug resistance-1) [27], which increase their resistance to several types of chemotherapeutic agents such as paclitaxel and 5-fluorouracil [27,28]. As cancer cells, the human TECs from patients are resistant to apoptosis, do not undergo senescence, and can grow under serum starvation [28].

At the molecular level, from a single-cell RNA sequencing (scRNA-seq) analysis conducted on TECs isolated from lung cancer patients, it emerged that, compared to NECs, TECs show a strong enriched signature in some signaling pathways such as the MYC pathway and the PI3K/Akt/mTOR pathway [29]. The pivotal role of MYC during both blood vessel formation in embryo development (vasculogenesis) and the angiogenic switch is supported by in vivo evidence [30]. It has also been proven that the PI3K/Akt/mTOR pathway plays a key role in tumor angiogenesis [31]. Surprisingly, Lambrechts and colleagues found a strong down-regulation of the inflammatory response in TECs [29].

A broader transcriptomic analysis, conducted on ECs from human (hTEC), murine (mTEC), and human cell culture (hcTEC) lung cancers, improved the molecular characterization of cellular phenotypes and identified new TEC markers in active sprouting [32]. It also emerged that the genes displaying higher expression in sprouting TECs are those encoding collagens (e.g., COL4A1, COL4A2, and COL18A1) and collagen-modifying enzymes (e.g., PXDN and PLOD1) [32]. This suggests that the extracellular matrix synthesis pathways, and in particular collagen synthesis, may represent a new target for striking growing tumor vessels.

## 2. Endothelial Metabolism during Angiogenesis

Adult vasculature is composed of quiescent ECs, also called phalanx cells. The quiescence state can be locally lost in favor of a proliferative state under pro-angiogenic stimuli. The current model of sprouting angiogenesis predicts that proliferating ECs sprout from a pre-existing vessel to anastomose with another newly formed sprouting vessel. Each sprout is guided by a tip cell, which is an activated EC cell, followed by so-called stalk cells [33]. This functional subdivision of ECs, based on the role played in angiogenesis, shows molecular and metabolic signatures [34].

Metabolism plays a crucial role in maintaining the quiescent state [35]. In particular, the forkhead box O (FOXO1) transcription factor has been reported as the gatekeeper of endothelial quiescence. FOXO1 suppresses the MYC pathway and down-regulates both glycolysis and oxidative phosphorylation (OXPHOS) [35]. Additionally, FOXO1 enhances branched-chain amino acid catabolites, shutting down ECs’ proliferation and angiogenesis in an MYC-independent manner [35]. Moreover, quiescent EC fatty acid consumption through β-oxidation is threefold greater than in proliferating ECs [36]. This is not related to the ATP production, which in any case remains mainly dependent on glycolysis, but serves as an anaplerotic reaction to fuel the NADPH production [36]. Understanding the molecular–metabolic mechanisms that trigger and maintain Ecs’ quiescence could provide a new toolbox for anti-angiogenic therapies. From this perspective, metabolically-induced quiescence could be used to impair tumor growth.

Following an imbalance between pro- and anti-angiogenic factors, phalanx cells can reactivate the cell cycle and become proliferating. Specialization in tip and stalk cells is a dynamic state that is dependent on receptors expressed on the EC surface [37]. The proliferating EC that most activates VEGF-VEGFR1/2 signaling is selected as the tip and, through overexpression in the NOTCH ligand DLL4, leads to the lateral inhibition of the other ECs, which then acquire the stalk phenotype [38]. Tip cells are highly dependent on glycolysis to produce the energy needed to support sprouting [9]. Despite the strong dependence on glycolytic flux, proliferating ECs cannot abolish OXPHOS. Respiratory chain complex III disruptions inhibits angiogenesis in vivo [39]. However, mitochondrial respiration reduces EC proliferation, but not migratory activity, which is the phenotypic hallmark of tip cells, suggesting a non-primary role of OXPHOS in tip–stalk differentiation. On the other hand, the endothelium-specific knockout of glutamine synthetase (GLUL) impairs angiogenesis by inhibiting migration [40]. Thus, the amino acid (AA) metabolism could be the main metabolic actor in promoting the tip identity.

## 3. Rewiring of Endothelial Metabolism in Cancer

### 3.1. Glycolysis

ECs utilize anaerobic glycolytic metabolism to produce most of the ATP rather than OXPHOS, regardless of living in an oxygen-rich environment [9]. Indeed, ECs produce around 80% of ATP via anaerobic glycolysis entering into the TCA cycle only 1% of glucose-derived pyruvate [41], lactate being the metabolic end-product of glycolysis in ECs. Under pro-angiogenic stimuli in a tumor context, the endothelial glycolytic rate increases and different glycolytic enzymes (e.g., hexokinase 2 (HK2) and pyruvate kinase (PK)) relocate into migratory cell structures, such as lamellipodia and filopodia, favoring cell migration [9]. Consistently, the endothelium-specific deletion of the *SLC2A1* gene, encoding glucose transporter type 1 (GLUT1), the main transporter of glucose uptake, reduces the formation of new blood vessels [42].

Analyses of scRNA-seq have shown that glycolysis-related genes are overexpressed, compared to NECs, in the transcriptomic signature of TECs (Table 1) [29,43]. Therefore, ECs within tumors appear to be more dependent on glycolytic flux (Figure 1B,C). Both genetic ablation and the pharmacological inhibition [44] of 6-phosphofructo-2-kinase/fructose-2,6-biphosphatase-3 (PFKFB3), a key enzyme in glycolysis regulation, inhibit blood vessel formation in tumor xenografts (Table 1). In addition, the PFKFB3 haplodeficiency or blockade induces tumor vessel normalization by tightening the EC barrier and promoting pericyte quiescence (Table 1) [11]. Moreover, FGF2, another proangiogenic factor, has been shown to increase glycolytic flux and regulate the glycolytic enzyme HK2 [45] via the MYC pathway [29]. It is interesting to note that OXPHOS factors are still expressed at high levels in TECs (Table 1) [29] and the inhibition of mitochondrial respiration prevents tumor angiogenesis [46]. These data reinforce the knowledge that OXPHOS serves to feed the growth of the tumor vasculature by anaplerosis more than producing energy.

While the effect of different glycolytic enzymes (e.g., PFKFB3, HK2, and PKM2) has been addressed in tumor angiogenesis, the role of glycolytic-branching pathways still remains largely unexplored, including hexosamine biosynthesis (HBP) and the pentose phosphate pathway (PPP) in an angiogenic cancer context. Although, it has been reported that there is an increased level of the PPP metabolite, sedoheptulose-7-phosphate (S7P) (Figure 1C), in TECs compared with NECs, but whether and how PPP is regulated during tumor angiogenesis is still unclear [11]. Moreover, recently a novel role of PPP in smooth muscle cell recruitment by regulating elastin production in zebrafish and mouse models in developmental angiogenesis has been published [47], but the potential role of this PPP controlling vessel normalization is yet to be elucidated. On the other hand, it has been described that the inhibition of HBP using azaserine increases flux throughout the TCA cycle and PPP, showing an interlink between these pathways in ECs [48], but the role of HBP in tumor angiogenesis remains unexplored.

**Table 1:** Direct in vivo evidence of TEC metabolic reprogramming that impairs tumor vasculature and consequently cancer growth.

Metabolic Pathway	Molecular Mechanism	Tumor Type	Reference
Glycolysis	PFKFB3 inhibition or haploinsufficiency normalize tumor vessel reducing glycolysis.	Melanoma, pancreatic tumor.	[11,44]
	Glycolytic genes over-expression.	NSCLC, Melanoma.	[29,43]
Lactate	VEGF-A induces carbonic anhydrase 2 (CAII), which reduces lactate acidosis in the tumor environment and enhances TEC survival.	Melanoma.	[49]
	Cancer-produced lactate enter ECs by monocarboxylate transporter (MCT-1) and fuels angiogenesis through NF-κB pathway, leading to the autocrine stimulation of IL-8.	Colorectal, breast cancer.	[50]
	Within TEC, lactate can be converted into pyruvate, which exerts a negative feedback on PHD2 triggering angiogenesis by HIF1α activation.	Pulmonary Lewis lung carcinoma.	[51]
OXPHOS	Mitochondrial respiration is necessary to sustain tumor angiogenesis.	Colorectal cancer, melanoma.	[46]
	OXPHOS genes over-expression.	NSCLC.	[29]
Hypoxia	EC-specific HIF1α deletion prevents autocrine signaling of VEGF, leading to reduction of vasculature.	Pulmonary Lewis lung carcinoma.	[52]
	PHD2 haploinsufficiency in ECs reduces glycolysis normalizing tumor vasculature.	Melanoma.	[53]
H_2_S	CBS silencing and pharmacological inhibition of CTH in cancer cells, the major H2S producers, reduce tumor angiogenesis.	Breast cancer.	[54]
	CTH knockdown reduces lymphangiogenesis.	Prostate cancer.	[55]
	CBS silencing reduces vasculature.	Colon cancer.	[55]
ECM stiffness	Pharmacological inhibition of the stiffness of the ECM reduces tumor angiogenesis.	Breast cancer.	[56]
	EC-specific knockout of YAP/TAZ results in impaired tumor and tumor vessel growth.	Melanoma.	[57]

### 3.2. Lactate

The high consumption of glucose followed by an incomplete oxidation within the tumor, known as anaerobic glycolysis, leads to the production of lactate. The release of lactate by tumor cells into the extracellular environment causes the acidification of TME and, thus, hypoxia and acidification always go together in tumors. The decrease in pH due to the accumulation of lactic acid, a toxic condition for NECs, stimulates TEC proliferation [49]. The expression of monocarboxylate transporter 1 (MCT1) in TECs allows to uptake lactate from the TME. Lactate flow in TECs activates the NF-kB pathway (Figure 1B), leading to the release of IL-8 that stimulates angiogenesis [50]. The pharmacological inhibition of MCT1 has been shown to reduce angiogenesis both in vitro and in vivo through the suppression of HIF-1α levels [51] (Figure 1B,C). This finding indicates that lactate is one of the driving forces of metabolic remodeling and tumor angiogenesis (Table 1).

### 3.3. Fatty Acid Oxidation

Lipids come from the diet or can be produced endogenously by the cells. In the endothelium, the blockade of both the lipogenesis [52] and lipolysis [53] cause angiogenesis defects. Specifically, ECs uptake fatty acids (FAs) by CD36 [54] and fatty acid transport proteins (FATPs) [55]. Pro-angiogenic signals, such as VEGF-B, increase the FAs flow by inducing the expression of FATPs [55], while the lymphatic endothelial cells (LEC)-specific deletion of CD36 alters the VE-cadherin junction and the integrity of the lymphatic vessels [56]. The accumulation of free FAs within the ECs triggers ROS generation compromising the integrity of the endothelium and triggering an inflammatory process [57]. Therefore, FAs should be continuously stored in membranes, degraded to produce energy and biomass, or esterified to proteins. Inside the cell, fatty acid-binding proteins (FABPs) bind and sort the FAs to the right compartment [58]. In ECs, VEGF signaling induces FABP4 expression [59], leading to vessel sprouting.

FAs are burned into the mitochondria through the fatty acid β-oxidation (FAO) pathway to generate energy and biomass. The rate-limiting step of this catabolic pathway is the import of FAs by carnitine palmitoyltransferase 1a (CPT1a) (Figure 1B,C). The endothelium-specific deletion of CPT1a reduces ECs proliferation [55]. Through the conditional knockout of endothelial carnitine palmitoyltransferase II (CPT2), an enzyme that works in synergy with CPT1a to regulate the carnitine shuttle, it has been shown that FAO is an endothelial-to-mesenchymal-transition (EndoMT) regulator [60]. The EndoMT of ECs promotes tumor aggressiveness by facilitating dissemination and rendering the vasculature insensitive to anti-VEGFR treatments [61]. Thus EC-specific up-regulation of the FAO pathway should antagonize EndoMT, ameliorating the survival rate. Thus, the up-regulation of the FAO pathway specifically in the ECs should antagonize EndoMT, ameliorating the survival rate.

### 3.4. Non-Essential Amino Acids

In recent years it has been become increasingly evident that amino acid (AA) metabolism also plays a pivotal role in regulating angiogenesis [62]. AAs had traditionally been classified as nutritionally essential (EAA, indispensable) or non-essential (NEAA, dispensable) depending on whether it is de novo synthesized or not. In this section we will discuss the new findings regarding tumor vasculature formation and NEAAs metabolism.

#### 3.4.1. Glutamine–Glutamate

Glutamine is the most abundant NEAA (~700 µM) in blood plasma being an indisputable source of nitrogen and carbon to support biosynthesis, energy, and anti-oxidant defense for the cellular homeostasis [63]. Despite that glutamine is a NEAA, ECs are not able to survive in absence of glutamine [64]. Indeed, ECs display a glutaminase (GLS) activity about 20-fold higher than lymphocytes, which have a high potential for rapid cell division [65]. Different works have described the crucial role of GLS in physiological angiogenesis showing that glutaminolysis fuels proliferation more than migration in ECs [64,66]. GLS blockade causes a drop of tricarboxilic acid (TCA) cycle intermediates and a subsequent decrease in macromolecular biosynthesis that leads to proliferation arrest [64,67].

Endothelial glutamine metabolism reprogramming has been related to different tumor extrinsic factors. Kaposi’s Sarcoma-associated Herpesvirus (KSHV), an oncogenic virus and the etiologic agent of Kaposi’s Sarcoma, induces glutaminolysis in ECs. Specifically, KSHV activates the MYC/MondoA-network to up-regulate the glutamine transporter, SLC1A5, leading to an increased glutamine up-take [68] (Figure 1B). Moreover, advanced tumors produce excessive amounts of TGFβ1, a cytokine associated with specific aspects of tumor progression including epithelial–mesenchymal transition (EMT), tissue invasion, and metastasis [69], which is able to induce GLS expression and promote endothelial cell sprouting by Raf/MEK/ERK activation [70]. Furthermore, it has been recently described that glutaminolysis drives tumor angiogenesis by controlling endothelial growth factor receptor 2 (VEGFR2) and fibroblast growth factor receptor 1(FGFR1) translation via mTORC1 activation [71] (Figure 1C).

Compared with glutamine, the concentration of glutamate in human plasma is lower (around 50 μM). Together with glycine and serine, glutamate is able to activate N-methyl-D-aspartate receptors (NMDAR) after binding the GRIN2D subunit, facilitating the cellular calcium influx [72] (Figure 1B). Interestingly, it has been shown that targeting GRIN2D in colorectal cancer by a vaccination approach leads to an inhibition of tumor growth and vascularization [73]. In addition, it has been shown that ECs utilize NMDAR1 for vasculature formation in gliomas, indicating a novel biological aspect of glutamate signaling in tumor angiogenesis [74]. Moreover, glutamate can be converted into glutamine by the glutamine synthetase enzyme (GLUL). ECs show negligible GLUL activity [68]. Eelen et al. have shown that the genetic deletion of *Glul* in ECs impairs vessel sprouting during vascular development, while minimally affecting healthy quiescent ECs. Mechanistically, *GLUL* knockdown reduces membrane localization and activation of the GTPase RHOJ, thereby inducing actin stress fibers and impeding endothelial cell motility [40]. Remaining unexplored is the role of GLUL in tumor angiogenesis.

#### 3.4.2. Aspartate–Asparagine

Aspartate participates in many reactions, including nucleotide and protein synthesis [75]. Due to its low concentration in blood (0–15 μM) [76], aspartate synthesis is crucial for cell survival. Aspartate biosynthesis is driven largely by glucose- or glutamine-dependent refilling of the TCA cycle to replenish mitochondrial oxaloacetate (OAA), which is subsequently converted to aspartate through the activity of mitochondrial glutamic-oxaloacetic transaminase 2 (GOT2). In humans, GOTs exist as two distinct isoenzymes: cytoplasmic GOT1 and mitochondrial GOT2. Both enzymes catalyze the same reaction albeit with different kinetics, share a sequence homology of ~45%, and are thought to have evolved from a common ancestral gene [76]. It is known that GOTs have multiple metabolic functions, including maintenance of the nicotinamide adenine dinucleotide/reduced nicotinamide adenine dinucleotide (NAD+/NADH) ratio in cells, α-keto acids production, gluconeogenesis, hydrogen sulfide (H_2_S) production via the CAT/3-mercaptopyruvate sulfotransferase pathway, and aspartate synthesis [77].

Even though the role of GOTs in physiological and pathological angiogenesis has been never investigated, several pieces of evidence suggest a crucial role of these enzymes in tumor angiogenesis. First, it has been shown that human recombinant VEGF-A induces the up-regulation of GOT2 in a study of chicken microarray analyses [78]. Second, an increased GOT2 level was addressed in angiogenic compared to non-angiogenic brain tumors in human glioblastoma xenograft studies [79]. Third, the endothelial-specific genetic ablation of mitochondrial complex III impairs tumor angiogenesis associated with a significant decrease in aspartate levels [39]. Fourth, it has been shown that short- and long-term hypoxia decreases aspartate availability in microvascular ECs [80]. Finally, the deletion of GOT1 in TECs blocks vessel formation in plug angiogenesis assays [71].

Asparagine, a proteogenic AA, is present in circulation (~100 μM) [81], and it can be synthesized by asparagine synthetase (ASNS), which converts aspartate and glutamine to asparagine and glutamate in an ATP-dependent reaction [82]. The enzyme is ubiquitous in mammals, but basal expression is relatively low in tissues other than the exocrine pancreas [83]. Human ASNS activity is highly regulated in response to cell stress, primarily by increased transcription [83]. ASNS expression and asparagine metabolism have received considerable attention in transformed cells, beginning with the observation that childhood acute lymphoblastic leukemia is susceptible to treatment by the infusion of bacterial asparaginase (ASNase) [84]. The role of asparagine metabolism has not been as extensively investigated in tumor angiogenesis. However, it has been shown that asparagine is crucial in glutamine-deprived ECs to reactivate mTOR signaling and protein biosynthesis [67].

#### 3.4.3. Serine

Serine is implicated in numerous metabolic processes such as antioxidant defense, one-carbon metabolism, and de novo nucleotide synthesis [85] (Figure 1C). Indeed, many cancers show an up-regulation of the biosynthetic enzymes involved in serine synthesis [86]. In fact, it has been reported that phosphoglycerate dehydrogenase (PHGDH) expression accelerates tumor growth in mouse models of melanoma and breast cancer; however, whether this acquired fitness advantage may induce the angiogenic process by secreting serine, remains obscure. Ten years ago, Maralani and colleagues showed that a serine pre-treatment (0.1–3.2 mM) protects ECs from hydrogen peroxide-mediated cell cytotoxicity and leads to significant induction of NRF2 activity, HO-1 expression, and NOx production [87]. Accordingly, it was reported that mouse neonates with EC-specific PHGDH deficiency suffer lethal vascular defects, due to reduced EC proliferation and survival. This phenotype is associated with insufficient heme production and consequently with elevated ROS levels due to cellular serine depletion [88]. In addition, recently it has been reported that dimethyl fumarate, a drug with known anti-angiogenic properties, inhibits the serine synthesis pathway by blocking PHGDH activity [89]. Despite serine having an active role in angiogenesis, its function in tumor angiogenesis is still poorly understood.

#### 3.4.4. Glycine

Glycine is involved in both angiogenesis and anti-angiogenesis. During angiogenic development, low concentrations of glycine promotes intersegmental vessel formation (ISVs), whereas high concentrations reduce angiogenesis in zebrafish embryos [90].

In a tumor context, glycine has been reported to suppress tumor angiogenesis. In fact, it was reported that glycine (100 μM) inhibited angiogenesis by more than 50% in a chorioallantoic membrane (CAM) assay. Dietary glycine supplementation reduces blood vessels in a fibrin Z-chamber assay and tumor angiogenesis in a tumor Z-chamber (fibrin with R3230 mammary adenocarcinoma cells) through the reduction of iNOS expression [91]. Moreover, tumor growth and vessel density were decreased in rats fed with 5% glycine compared without it in a WAG-Rij/CC-531 rat model of metastatic colorectal cancer [92]. On the other hand, glycine promotes angiogenesis in other pathological contexts. For example, in a hindlimb ischemia model, glycine treatment significantly enhanced neovascularization promoting the recovery of vascular flow via the GlyT1-glycine-mTOR-VDAC1 axis pathway [93]. Furthermore, glycine prevents the apoptosis of rat sinusoidal endothelial cells caused by Blc-2 decrease following VEGF deprivation [94]. The seemingly inconsistent results of these studies on glycine may be related to differences in the species, tissues, and cells used, or may be attributed to different approaches (e.g., culture conditions, doses and time of stimulation, animal models, and disease models). However, we cannot discard glycine as a novel target for angiogenic and anti-angiogenic therapy.

#### 3.4.5. Cysteine

Cysteine is a nutritional semi-essential AA that is present mainly in the form of cystine (Cys–Cys dimmer) in the extracellular space [95,96]. The maintenance of relatively low concentrations of cysteine in the body is critical because cysteine is toxic at high levels. Yet, the level of cysteine must be high enough to allow its use for other purposes, such as the formation of glutathione (GSH) and the synthesis of proteins (Figure 1C) [97]. In culture, cysteine seems to be indispensable for EC growth and survival [98]. The uptake of cystine in ECs is Na^+^-independent and inhibited competitively by glutamate being mediated by system xCT [99]. Recently, it has been revealed that xCT and its substrate glutamate specifically operate on ECs and promote neoangiogenesis [74]. Thus, targeting xCT expression and glutamate secretion in gliomas provides a novel therapeutic roadmap for normalizing tumor angiogenesis [74]. In addition, EC treatment with erastin, an inhibitor of xCT, at a non-lethal level promotes EC proliferation, migration, and vessel-like structures formation, concomitantly with a reduction of GSH and an increase in ROS. The dual therapy using propranolol, which reverts the erastin-dependent activation of ECs, and chrysin to induce cytotoxic to cancer cells, has been proposed [100].

On the other hand, the heterodimeric transporter LAT1/4F2hc mediates the transport of essential (histidine, isoleucine, methionine, tryptophan, phenylalanine, leucine, cysteine, and tyrosine) and non-essential (glutamine) AAs across the cell membrane, the affinity of EAAs for LAT1 being higher compared NEAAs [101]. In human pancreatic ductal adenocarcinoma xenograft mouse models, a significant increase in LAT1 expression in TECs compared with NECs has been reported [102]. Molecularly, LAT1 is crucial to support angiogenesis-mediated amino acid transport, which is indispensable for the VEGF-A-dependent activation of mTORC1.

Cysteine seems to be an indispensable AA for ECs survival, reflecting a high requirement for antioxidants to protect ECs from oxidative stress [98]. In addition, it has been described that in tumor cells, cysteine activates mTORC1 through the GCN2/ATF4/SESN2 axis, inducing cell growth [103]. The replenishment of cellular GSH with thiol-amino acids counteracts the growth-inhibitory effect of TGF-β1 in ECs through a currently undefined mechanism [104]. These findings support the crucial role of cysteine in both the survival and growth of ECs.

#### 3.4.6. Tyrosine

Mammals synthesize tyrosine from the EAA phenylalanine in a reaction catalyzed by phenylalanine hydroxylase. This enzyme hydroxylates phenylalanine to tyrosine and is the rate-limiting step in phenylalanine catabolism. Phenylketonuria (PKU) is an autosomal-recessive inborn error of phenylalanine (Phe) catabolism, caused by the deficiency of phenylalanine hydroxylase [105]. It has been reported that patients with PKU show endothelial dysfunction [105] and increased aortic stiffness compared to healthy controls [106], changes that could be associated with a secondary deficit in tetrahydrobiopterin (BH4) (Figure 1C) [107]. Consistently, the oral supplementation with Phe is able to enhance endogenous BH4 biosynthesis through the GCH1–GFRP protein complex, elevates nitrite levels, reduces vascular ROS levels, and improves endothelium-dependent vascular relaxation in a spontaneously hypertensive rat model [108]. Furthermore, it has been demonstrated that BH4 synthesis via either the *pterin salvage* or the de novo pathway, promotes endothelial cell proliferation, migration, and tubule formation in cultures and induces angiogenesis in tumor xenografts [108].

### 3.5. Metabolic Interactions between Tumor Tissue and Endothelial Cells

Tumors exhibit an altered metabolism compared to non-transformed tissues [109]. The discovery and characterization of tumor reprogrammed metabolisms may provide opportunities to predict endothelial behavior and prevent tumor angiogenesis by targeting tumor–endothelial metabolic interactions.

Symbiotic and competitive metabolic interactions between tumor cells and microenvironmental cells have been reported in various cancers. For example, pancreatic stellate cells (PSCs) utilize SLC1A4 to exchange and maintain extracellular alanine levels. Moreover, pancreatic ductal adenocarcinoma (PDAC) cells up-regulate SLC38A2 and increase their alanine demand. PDACs lacking SLC38A2 fail to concentrate intracellular alanine and undergo a profound metabolic crisis resulting in a tumor growth impairment [110]. In addition, metabolites profiling of melanoma interstitial fluids reveals uridine diphosphate as a potent immune modulator capable of limiting tumor growth by increasing CD4^+^CD25^+^FoxP3^−^ cells [111]. Lastly, targeting GLUL in cancer-associated fibroblasts induces tumor regression in an orthotopic ovarian cancer model [112]. However, it remains unanswered whether it could be possible target stromal metabolic pathways to find therapeutic opportunities to block tumor angiogenesis.

Recently, the impact of breast cancer subtypes on LEC metabolism and lymphangiogenesis was studied. Specifically, LECs co-cultured with breast cancer cell lines increased glycolysis. Moreover, lactate levels were significantly increased, which correlated to the up-regulation of lactate metabolism enzymes and transporters [113].

Emerging data have identified cooperative and competitive relationships between cancer cells and TECs. Indeed, it has been shown that blocking the heme exporter Feline Leukemia Virus subgroup C Receptor 1a (FLVCR1a) in TECs causes ketone bodies (KBs) accumulation. TEC-derived KBs can be secreted producing a metabolic rewiring in the cancer cells [114]. In addition, it has been reported that tumor-associated macrophages (TAMs) can up-regulate the expression of REDD1, a negative regulator of mTOR, under hypoxic conditions. REDD1-deficient TAMs are highly glycolytic cells that out-compete ECs for glucose usage, which thwarts vascular hyperactivation and promotes the formation of quiescent vascular junctions. Tuning down glycolysis in REDD1 knockout TAMs re-establishes abnormal angiogenesis and metastases [115].

### 3.6. Gasotransmitters: Signals in the Air

Not all biologically active molecules have a solid nature. In fact, there are volatile molecules capable of arousing functional responses in cells and tissues. These molecules, known as gasotransmitters, can transduce signals and therefore influence cell activities [116]. An increasing body of evidence is starting to shed light on the roles played by gasotransmitters, for example in the tumor context [117]. It is interesting to note that all known gasotransmitters are in some way linked to the physiology of the vascular endothelium. This is not surprising since the vascular system has evolved to facilitate gas exchange between tissues, but this makes ECs an excellent model for investigating and dissecting the gasotransmitter pathways. Here, we will briefly discuss the role of major gasotransmitters on endothelial cell metabolism, a new emerging, and still largely unexplored field.

Nitric oxide (NO) is a gas produced by enzymes called nitric oxide synthases (NOSs), which convert arginine into citrulline in a reaction involving oxygen and various coenzymes such as NADPH and BH4. The NOS family contains neuronal NOS (nNOS/NOS1), inducible NOS (iNOS/NOS2), and endothelial NOS (eNOS/NOS3). In ECs, both eNOS and iNOS are under the control of VEGF [118]. eNOS full knockout mice show a strong impairment in vascular development [119]. Gene therapy aimed at overexpressing eNOS in an ischemic hindlimb rat model improves the perfusion of the tissue [120]. NO produced by NOS acts in an autocrine and paracrine manner on the cells of the vascular system leading to the direct activation of some signaling pathways [121] or through post-translational modifications such as S-nitrosylation [122]. In ECs, the S-nitrosylation reduces the reserve respiratory capacity, while stimulating glycolysis [123] (Figure 1B). The supplementation of ECs with NO-donors leads to an increase in glucose uptake coupled to GLUT1 up-regulation, and enhances HK activity [124]. This indicates that S-nitrosylation, NO-induced, acts not only as an actor of metabolic reprogramming, but also as a possible driver of glycolytic metabolism during angiogenesis. However, it has recently been found that eNOS inhibits the glycolytic enzyme PKM2 through S-nitrosylation, which leads to an increase in PPP activity to generate reducing equivalents. By this mechanism, NO protects the vasculature from oxidative stress and exerts anti-inflammatory effects [125].

Another gasotrasmitter is the hydrogen sulphyde (H_2_S). H_2_S is an endogenous product of sulfur AA metabolism (Figure 1B). It is released during the transsulfuration pathway (TSP), the metabolic pathway that leads to the de novo synthesis of cysteine from the methionine by cystathionine β-synthase (CBS) and cystathionine γ-lyase (CTH) [126]. Cancer cells express high levels of CBS and CTH and their genetic or pharmacological inhibition leads to a reduction in tumor vasculature [125,126,127], suggesting that the TSP metabolic reprogramming may be a promising therapeutic strategy for solid tumors (Table 1). Finally, in ECs, H_2_S enhances glucose uptake and glycolytic ATP production, and at the same time inhibits the complex IV (Figure 1B), leading to the reduction of mitochondrial OXPHOS [128]. Taken together, these data indicate TSP as a powerful driver of angiogenesis and a putative target for anti-angiogenic therapy.

Not only can the presence of a gas have biological effects on metabolism, but also its shortage. The best known and studied case is the lack of oxygen (O_2_), or hypoxia. All multi-cellular living organisms consume O_2_ to support their own energy request. However, when in each tissue, O_2_ level-decreased cells must adapt their metabolic needs to new conditions. At the molecular level, the cell senses and transduces the signal through the hypoxia-inducible factor pathway [129]. HIF-1α deletion in ECs impairs vessel growth within cancers [129], highlighting the essential role of hypoxia in tumor angiogenesis. Surprisingly, PHD2 haploinsufficiency in ECs bears tumor vascular normalization, resulting in a better oxygenation and metastases reduction in a tumor xenograft model [130]. The transcriptomic analysis of hypoxic ECs has shown the importance of HIF-1α in up-regulating the glycolytic factors and down-regulating the mitochondrial metabolism [131]. In addition, glycolysis-associated genes such as SLC2A3, PFKFB3, and HK2 belong to the early hypoxia-responsive genes [78,131].

### 3.7. Extracellular Matrix Stiffness and Angiogenesis

The niche in which tumors develop and grow often becomes a fibrotic and extremely rigid microenvironment, referred as desmoplastic stroma. The stiffness is associated with the cancer aggressiveness [132]. This stiffness enhancement is due to the progressive remodeling of the extracellular matrix (ECM) induced by the tumor [132,133]. In addition, to being embedded in a rigid desmoplastic stroma, highly aggressive tumors are usually highly vascularized [3]. Consistent with this, it has been reported that the ECM stiffening is able to trigger tumor angiogenesis (Figure 1B) [134]. The mechanical forces acting inside a tumor play a central role in the formation of new vessels and can act in different ways. For example, it has been reported that mechanical tension is able to induce the activation of the VEGFR2 [135]. While the pressure induced by the stiffening of the matrix causes the destruction of the cell–cell endothelial junctions [134], leading to the release and activation of YAP/TAZ that regulates both physiological and tumor angiogenesis [136] (Table 1). These factors are downstream targets of the VEGF pathway and are necessary to induce the migratory phenotype in sprouting ECs [137,138]. Furthermore, a proteomic analysis based on quantitative mass spectrometry showed how ECs cultured into different matrix stiffnesses promote metastasis through a CCN1-dependent mechanism [139]. CCN1 signaling promotes the adhesion between endothelial and tumor cells by inducing the expression of N-cadherin [139], thus facilitating the dissemination of cancer cells. It is interesting to note how during embryonic development the activation of YAP regulates GLUT1/2 expression, increasing the consumption of glucose and nucleotides synthesis [140]. While in breast cancer cells, matrix stiffness causes a metabolic shift from a predominantly glycolytic to OXPHOS and FAO metabolism [141]. On the other hand, in pulmonary arterial ECs and smooth muscle cells, an increase in glycolysis coupled by an increase in glutaminolysis to fuel proliferation was observed [142]. This strongly suggests that matrix stiffening can influence the metabolic reprogramming of ECs. Despite both the role of stiffness and metabolism in promoting angiogenesis being widely documented, there is still a lack of knowledge that connects these two fundamental aspects in the tumor context.

Another mechanical stress to which TECs are subjected is an irregular and turbulent flow that reduces tumor perfusion, increasing its malignancy. In general, the laminar flow into the adult vasculature helps to keep ECs quiescent by activating the cell cycle checkpoints through the induction of p53 and p21 [143]. A disturbed flow activates the mechano-transducers YAP/TAZ, promoting their dephosphorylation and translocation into the nucleus, where they propel proliferation and an inflammatory phenotype [144]. A low fluid shear stress promotes the adhesion and extravasation of cancer cells to the vascular endothelium [145]. Instead, a physiological-like flow activates the pro-quiescence factor KLF2, which represses PFKFB3 gene expression and inhibits glycolytic metabolism. However, it has been reported that an increased laminar flow in circulating cancer cells enhances the transcription factor ATOH8, which, in turn, induces the expression of the glycolytic enzyme HK2 [146]. This confers a proliferative advantage on metastatic cells. Laminar flow appears to play a dual role in tumor progression, therefore further investigations are needed to better dissect the mechanisms induced on the metabolism of endothelial and tumor cells.

## 4. Conclusions and Perspectives

Altogether these findings indicate that researchers are moving away from the only VEGF-oriented model of angiogenesis, and start to realize that endothelial metabolism cooperates with canonical signaling mechanisms to drive vascular morphogenesis and differentiation. The next challenge will be to reveal the functional crosstalk among all these conditions in normal and pathological conditions. This aspect is intriguing considering that most metabolic pathways regulate the classical angiogenic signaling pathways such as VEGF, FGF, or NOTCH signaling. Novel technologies, such as metabolic sensors (e.g., in vivo metabolite tracers) and single cell technology coupled to system biology are needed to spatially and temporally decode these mechanisms and comprehend how angiogenic signal networks are regulated in diseased angiogenic conditions. We strongly believe that all these aspects will lead to important and unforeseen advances in the tumor angiogenic field in the coming years.
